# Efficacy of low-dose radiotherapy in painful gonarthritis: experiences from a retrospective East German bicenter study

**DOI:** 10.1186/1748-717X-8-29

**Published:** 2013-01-31

**Authors:** Stephanie Keller, Klaus Müller, Rolf-Dieter Kortmann, Ulrich Wolf, Guido Hildebrandt, André Liebmann, Oliver Micke, Gert Flemming, Dieter Baaske

**Affiliations:** 1 Department of Internal Medicine II- Gastroenterology / Hepatology / Oncology / Infectology / Tropical Medicine /Endocrinology / Diabetology, Klinikum Chemnitz gGmbH, Flemmingstraße 2, 09116, Chemnitz, Germany; 2 Department of Radiotherapy, University Hospital of Leipzig, Stephan-Str. 9a, 04103, Leipzig, Germany; 3 Department of Radiotherapy, University Medicine Rostock, Südring 75, 18059, Rostock, Germany; 4 Department of Radiotherapy, Franziskus Hospital Bielefeld, Kiskerstraße 26, 33615, Bielefeld, Germany; 5 Department of Radiology, Heliosklinikum Aue, Gartenstraße 6, 08280, Aue, Germany; 6 Department of Radiotherapy, Klinikum Chemnitz GmbH, Flemmingstraße 2, 09116, Chemnitz, Germany

**Keywords:** Osteoarthritis, Knee, Radiotherapy, Irradiation, X-ray, Gonarthritis, Gonarthrosis, Osteoarthrosis

## Abstract

**Purpose:**

To evaluate the efficacy of low-dose radiotherapy in painful gonarthritis.

**Methods:**

We assessed the medical records of 1037 patients with painful gonarthritis who had undergone low-dose radiotherapy between 1981 and 2008. The subjective patient perception of the response to irradiation as graded immediately or up to two months after the completion of a radiotherapy series was evaluated and correlated with age, gender, radiological grading and the duration of symptoms before radiotherapy. Moreover, we performed a mail survey to obtain additional long-term follow-up information and received one hundred and six evaluable questionnaires.

**Results:**

We assessed 1659 series of radiotherapy in 1037 patients. In 79.3% of the cases the patients experienced a slight, marked or complete pain relief immediately or up to two months after the completion of radiotherapy. Gender, age and the duration of pain before radiotherapy did not have a significant influence on the response to irradiation. In contrast, severe signs of osteoarthritis were associated with more effective pain relief. In more than 50% of the patients who reported a positive response to irradiation a sustained period of symptomatic improvement was observed.

**Conclusions:**

Our results confirm that low-dose radiotherapy is an effective treatment for painful osteoarthritis of the knee. In contrast to an earlier retrospective study, severe signs of osteoarthritis constituted a positive prognostic factor for the response to irradiation. A randomized trial is urgently required to compare radiotherapy with other treatment modalities.

## Background

Painful osteoarthritis of the knee is one of the world’s most common degenerative joint disorders [1,2]. Its analgetic treatment with low-dose ionizing radiation has a long tradition in Germany [3-5]. Nevertheless, the acceptance of this method, especially abroad, has to be considered low. Accordingly, radiotherapy still has not been included as a therapeutic option in the European League against Rheumatism (EULAR) guidelines [6] and usually represents the “last resort” before surgery. Until recently the pain-relieving effect of radiotherapy in painful gonarthritis was mainly demonstrated by earlier studies, which were highly susceptible to criticism due to methodological weaknesses, outdated radiation techniques and partly low numbers of patients. In 2010 the German Cooperative Group on Radiotherapy for Benign Diseases (GCG-BD) resumed the issue again releasing the results of a large pattern of care study on the role of radiotherapy in painful and refractory gonarthritis which had been conducted in 42 German radiotherapy institutions from 2006 to 2008. High response and low toxicity could be demonstrated in a very large number (n = 5069) of cases [5]. However, one may object that there are methodological problems that are inherent to this kind of survey. The quality of data can be verified only with great difficulty and the information may reflect rough estimates or personal opinions. The purpose of this retrospective bicenter study was to provide evidence that radiotherapy is effective in the treatment of painful gonarthritis and thus can be a reasonable alternative to other therapeutic options. Furthermore we investigated the influence of possible prognostic factors on the pain relieving effect of radiotherapy. We finally performed a systematic literature search and discussed our results in the light of recent releases.

## Methods

The clinical data of 1037 patients who had undergone radiotherapy for painful gonarthritis in the hospitals of Chemnitz and Aue between 1981 and 2008 were evaluated retrospectively. The diagnosis was based on medical history, orthopaedic examination, which was performed by the referring physician and/or the radiotherapist and a conventional X-ray examination. The classification of the radiological severity of gonarthritis in “normal”, “minimal”, “moderate” and “severe” was made by entries in the patient files using the Kellgren-Lawrence score [7]. Most of the patients were treated using orthovoltage units with a radiation of 150, 175, 180 or 200 kV, 20 mA and copper filters of 0.5 or 1.0 mm thickness. The focus-skin distance was 40 or 50 cm. Some patients were treated on a linear accelerator with 6 or 9 MeV photons or on a radiotherapeutic unit with a Cs-137 radiation source in the 1980s. The joints were irradiated from medial and lateral and in some exceptional cases from anterior and posterior with field sizes ranging between 8 x 10 and 15 x 20 cm^2^. Radiotherapy was performed once a week in 611/1659 series (36.8%), twice a week in 1045/1659 series (63.0%) or daily in 3/1659 series (0.2%). The single (total) doses ranged between 0.5 and 1.5 Gy (0.5 and 10 Gy) for a series (Table 1). As a matter of common practice two different radiotherapy techniques were applied. In Aue two opposed irradiation fields were used and the reference point of the dose was placed on the skin surface. In Chemnitz the knee joints were irradiated using a lateral and a medial (or a ventral and a dorsal) field in alternation and the reference point of the dose was placed in the center of the joint. The response to treatment was recorded, as it was subjectively graded by the patient immediately or up to two months after completion of treatment. Improvement of pain was categorized based on the classification published by von Pannewitz in 1933 [8] (painless, markedly improved, improved, stable, worse). In order to complete missing follow-up information questionnaires were mailed to 248 patients who had been treated in Chemnitz between 1996 and 2008. One hundred and six evaluable questionaires (42.7%) were finally returned. The questionnaires addressed the pain relief after treatment in a four-stage classification, the ability to move in a three-level classification and the period during which a possible improvement had been observed.

**Table 1:** Summary of the fractionation schemes & dose prescriptions

**Item**	**N=**	**%**
**Fractions per week**		
1	1045	63.0
2	611	36.8
5	3	0.2
**Single dose (Gy)**		
0,5	248	14.9
1	1389	83.7
Other	22	1.3
**Total dose (Gy)**		
4	899	54.2
6	122	7.4
Other	638	38.5
**Total**	1659	100

In 1037 patients with painful osteoarthritis of the knee who underwent radiotherapy (1659 series) between 1981 and 2008.

### Statistical analysis

Potential prognostic factors as age, gender, radiological grading and the duration of pain before radiotherapy were correlated with the response to irradiation using the chi square test. Statistical analysis was performed using MS Excel 2007 and SPSS 15.0.

### Literature search

We performed Pubmed and Web of Science search with predefined search terms. The search was limited to English- and German-language articles published since 1980. English key words were: osteoarthritis, knee, radiotherapy, irradiation, x-ray, gonarthritis, gonarthrosis, osteoarthrosis. German key words were: Osteoarthrose (itis), Arthrose (itis), Knie, Röntgenreizbestrahlung, Bestrahlung, Radiotherapie, Gonarthrose (itis). We identified 109 articles and abstracts by the search terms. After title and abstract review a preclinical [9] and four clinical articles [5,10-12] about radiotherapy in painful gonarthritis were eligible. In addition, we performed hand search following the references from selected articles.

### Ethical principles

This retrospective study is in compliance with the Declaration of Helsinki - Ethical Principles for Medical Research Involving Human Subjects – and its amendments.

## Results

### Patient characteristics

All in all 1659 series of radiotherapy in 1037 patients were assessed (Table 2). Three hundred sixteen patients were male (30.5%) and 721 female (69.5%). The age at the beginning of the first radiotherapy series ranged from 23 to 93 years. Patients younger than 40 years accounted for only a small proportion of the whole cohort (n = 15/1037, 1.5%). Usually this group included patients who were suffering from post-traumatic osteoarthritis and had severe pain. The group of fourty to sixty-year-old was represented by a total of 304 patients (29.3%). The group of over-sixty-year-old constituted the majority of patients (n = 718/1037, 69.2%). 662 patients (63.8%) underwent only a single series. 237 patients (22.9%) received two, 84 patients (8.1%) three and 54 patients (5.2%) more than three series. On average, each patient was irradiated with 1.6 series. 11.4% of the patients who presented more than once were treated on both knees. Women were significantly more likely to undergo various series of radiotherapy on the same knee than men. 42.6% of the women were treated with two or more irradiation series, for men, the proportion was 37.6%. In the majority of cases pain symptoms had occurred more than three years prior to the first irradiation (n = 439/1037, 42.4%). Twenty-one percent of the patients each suffered pain for less than a year (n = 213/1037) and for one to three years (n = 215/1037) respectively. Information about the radiological severity of arthritis before the first irradiation was available in 471 joints, which were irradiated with 654 series. 228 of these 654 series were effected to knees with moderate osteoarthritis. The knees with radiologically proven severe osteoarthritis received 308/654 series. In 119/654 series the joints showed minimal signs of osteoarthritis. 3/654 series were effected to joints that were normal on imaging. 4.2% of the treated knees depicted axial deformity. The varus deformity was described nearly five times more often than the valgus deformity (82.5% versus 17.5%). A traumatic pre-damage of the knee cold be elicited in 12.5% of the irradiated knees. Overall, 63.9% of the patients reported the application of alternative pain-killing treatments before the first radiotherapy series. The intra-articular administration of corticosteroids was first in line.

**Table 2:** Patient characteristics

**Item**	**N=**	**%**
**Gender**		
Male	316/1037	30.5
Female	721/1037	69.5
**Age group**		
≤ 60 years	319/1037	30.8
> 60 years	718/1037	69.2
**Severity**		
Minimal	119/651	18.3
Moderate	228/651	35.0
Severe	304/651	46.7
**Duration of pain**		
< 1 year	213/867	24.6
1 – 3 years	215/867	24.8
> 3 years	439/867	50.6

Clinical data of 1037 patients with painful osteoarthritis of the knee who underwent radiotherapy between 1981 and 2008.

### Response to treatment as recorded immediately or up to two months after radiotherapy

**Figure 1:**
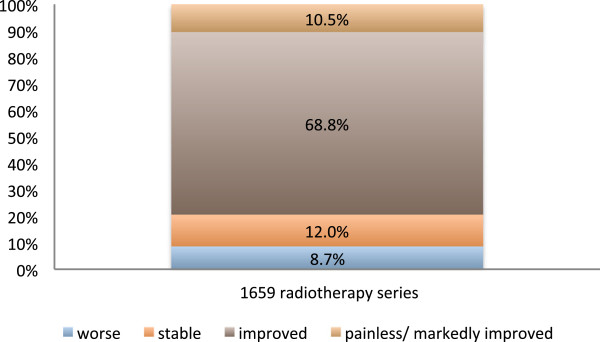
**Overall response to radiotherapy.** Pain, as it was subjectively graded by the patients immediately or up to two months after the completion of a series of radiotherapy.

**Figure 2:**
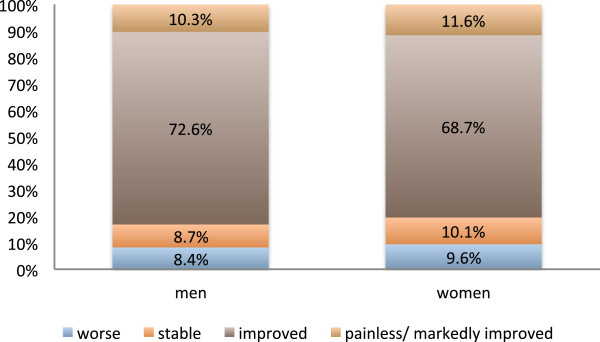
**Response to radiotherapy split by gender.** Pain, as it was subjectively graded by the patients immediately or up to two months after the completion of a series of radiotherapy. There was no significant difference in radiation-induced pain relief between men and women (p = 0.347).

**Figure 3:**
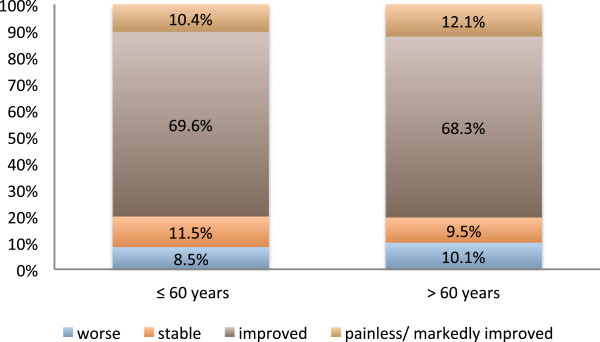
**Response to radiotherapy split by age.** Pain, as it was subjectively graded by the patients immediately or up to two months after the completion of a series of radiotherapy. There was no significant difference in radiation-induced pain relief between under- and over-sixty-year-old patients (p = 0.505).

**Figure 4:**
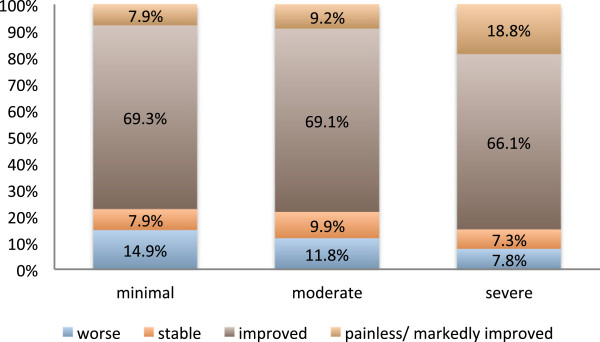
**Response to radiotherapy split by radiological severity of gonarthritis.** Pain, as it was subjectively graded by the patients immediately or up to two months after the completion of a series of radiotherapy. There was a significant difference in radiation-induced pain relief between minimal/ moderate and severe gonarthritis (p = 0.036).

**Figure 5:**
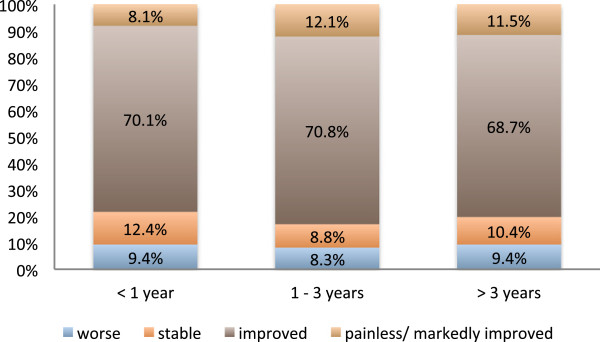
**Response to radiotherapy split by duration of pain prior to treatment.** Pain, as it was subjectively graded by the patients immediately or up to two months after completion of a series. There was no significant difference in radiation-induced pain relief between the patients who reported a history of pain of less than a year, 1 to 3 years or more than 3 years prior to radiotherapy (p = 0.699).

Reliable short-term follow-up information concerning pain relief was available in 1659 radiotherapy series. In 79.3% of the cases patients were painless or experienced marked or at least slight pain relief immediately or up to two months after the completion of radiotherapy (Figure 1). Neither gender (Figure 2) nor age (cutoff 60 years) (Figure 3) had a significant influence on the pain-relieving effect. Figure 4 refers to 471 joints with complete information on the radiological severity of osteoarthritis prior to irradiation. In 84.9% of the cases with severe signs of osteoarthritis radiotherapy led to slight or marked pain-relief. In the cases with minimal / moderate signs of osteoarthritis this result was only achieved in 77.2% / 78.3%. The difference proved to be statistically significant (p < 0.05). There was no significant difference in radiation-induced pain relief between the patients who reported a history of pain of less than a year, 1 to 3 years or more than 3 years prior to radiotherapy (Figure 5).

### Response to treatment as recorded by the additional mail survey

**Figure 6:**
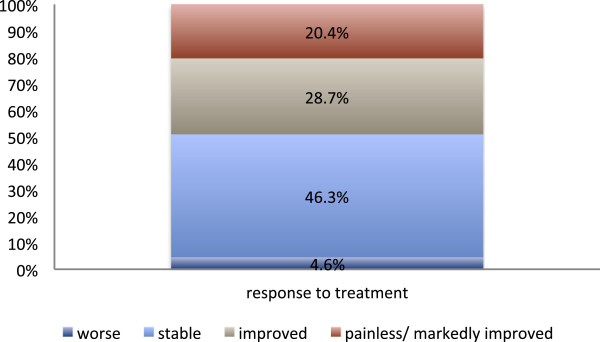
**Response to radiotherapy according to the additional mail survey (106 evaluable questionnaires).** Pain after the end of radiotherapy, as it was subjectively graded by the patients in a retrospective mail survey, which was effected in 2010, i.e., two to fourteen years after treatment.

**Figure 7:**
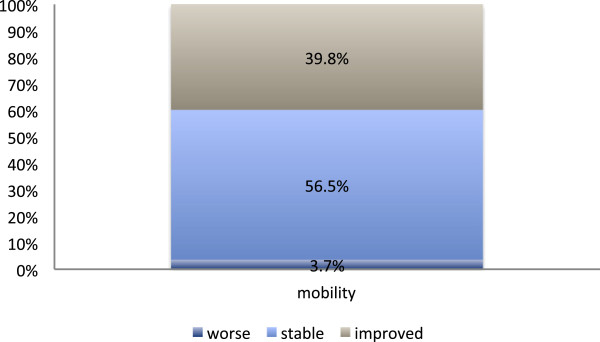
**Response to radiotherapy according to the additional mail survey (106 evaluable questionnaires).** Mobility after the end of radiotherapy, as it was subjectively graded by the patients in a retrospective mail survey, which was effected in 2010, i.e., two to fourteen years after treatment.

**Figure 8:**
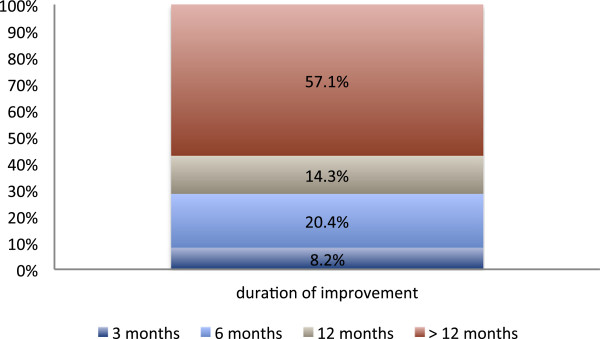
**Response to radiotherapy according to the additional mail survey.** Duration of clinical improvement after radiotherapy, as it was subjectively reported by the patients in a retrospective mail survey, which was effected in 2010.

It was found that a total of 49.1% (39.8%) of the respondents had indicated a beginning or significant pain relief (mobility improvement) after treatment (Figure 6 & Figure 7). In more than 50% of the patients who reported a positive response to irradiation a sustained period of symptomatic improvement was observed (Figure 8).

## Discussion

### Best response to radiotherapy

Similar to the previous studies identified by our literature search this large retrospective analysis demonstrates the good analgetic effect of radiotherapy in painful osteoarthritis of the knee. In 80% of the cases the irradiated patients reported a positive response to treatment immediately or up to two months after the completion of the respective series. However, the proportion of very good responders, i.e., patients who were free of pain or in whom the symptoms had markedly improved after a series of radiotherapy, was low in comparison to the other series (Additional file 1: Table S1). A possible explanation for that phenomenon could be that, for practical reasons, the response to treatment had been evaluated more likely immediately rather than several weeks after the completion of radiotherapy.

### Point of time of the best response to radiotherapy

The assessment of the treatment effect should be performed immediately and a few weeks after the completion of radiotherapy, since in a high percentage of cases outcome apparently improves over time. Several analyses demonstrate that approximately ten percent of the patients are free of symptoms or feel significant pain relief directly after the completion of radiotherapy [10,13]. In the cohort assessed by Keinert et al. the proportion of patients with complete resolution of symptoms increased from 8 to 38 percent in the following six weeks [10]. In a cohort of 21 patients assessed by Sautter-Bihl et al. the final treatment success occurred in 10% during, in 14% immediately after and in 76% within six weeks after radiotherapy [13]. Accordingly Ruppert et al. investigated a cohort of 73 patients with painful osteoarthritis affecting different joints and reported a delay of ten weeks between the start of radiotherapy and by pain reduction in 47% of the patients who responded to treatment [12].

### Duration of the analgetic effect

57% of the responders in our cohort experienced notable pain-relief for more than a year (Figure 8). In contrast, Mücke et al. reported a median estimate for the share of patients who experienced pain reduction for at least 12 months of only 40% [5]. In the cohort of patients assessed by Sautter-Bihl et al. the pain-relieving effect of radiotherapy lasted longer than 12 months in 67% of the cases [13]. In the analysis performed by Keinert et al. the share of the responders who suffered a recurrence ranged between 30% (patients with marked pain-relief) and 50% (patients who were free of pain after radiotherapy) [10]. This observation was confirmed by Keilholz at al. who found a relapse rate of 33% in a cohort of 30 responders [11]. The analgetic potential of radiotherapy in painful osteoarthritis of the knee seems to be comparable to that in other big joints [12].

### Prognostic factors

#### Gender and age

Neither gender (Figure 2) nor age (Figure 3) had a significant influence on the pain-relieving effect in this retrospective study. In contrast in the analysis performed by Keilholz et al. the patients < 80 years tended to have a more favorable treatment response (univariate analysis, p = 0.08) [11]. However this trend could not be confirmed in multivariate analysis. In the study of Glatzel et al. age (≤ 60 years versus > 60 years) did not influence outcome whereas best results with a clear analgesic effect were reached in males (29/50, 58% versus femals: 39/135, 29%). The factor gender had an independent prognostic value (p < 0.01) [14].

#### Severity

The impact of the radiological severity of osteoarthritis on the response to radiotherapy remains controversial. In this study in 84.9% of the cases with severe signs of osteoarthritis the first irradiation series led to slight or marked pain-relief. In the cases with minimal/ moderate signs of osteoarthritis this result was only achieved in 77.2%/ 78.3%. The difference proved to be significant. In contrast, in the analysis released by Keilholz et al. the patients with severe radiological signs of osteoarthritis tended to respond worse to radiotherapy in univariate analysis [11].

#### Duration of symptoms

Some authors stated that the results of treatment were dependent on the duration of symptoms before the start of radiotherapy. In the cohort of Keinert et al. 48% of the patients with a short duration of symptoms (<1 year) were pain-free after irradiation. In patients with a longer duration of symptoms (>1 year) this result was only achieved in 25%. Keinert et al. derived from their observations the demand to use irradiation, contrary to the usual practice, as early treatment option [10]. This demand was supported by Keilholz et al. and Glatzel et al. who demonstrated that a short duration of pain symptoms before the start of radiotherapy (≤ 2 years and ≤1 year respectively) was an independent positive prognostic factor for the success of pain-relieving radiotherapeutic treatment in multivariate analysis (p < 0.05) [11,14]. In contrast, in our cohort there was no significant difference in radiation-induced pain relief between the patients who reported a history of pain of less than a year, 1 to 3 years or more than 3 years prior to radiotherapy.

#### RT–technique and dosage

The optimal radiotherapy technique and dosage are currently unknown. Frequently two opposed irradiation fields are used and the reference point of the dose is placed in the center of the joint. However, assuming a typical knee diameter of 10 cm, the clinical relevance of this approach may be questioned, as the respective dose distributions within the knee joint will diverge only marginally if the reference point is shifted to the skin surface (Additional file 1: Figure S1). Although in our cohort the use of alternating single fields was associated with a better response to treatment (data not shown), we are reluctant to derive radiotherapy planning recommendations from those findings as our results may be biased by the variety of additional factors influencing the dose distribution within the knee joint (i.g. acceleration voltage, thickness of the filter, focus-skin distance, field size, diameter of the knee) by different dose prescriptions and fractionation schemes (Table 1) or other not treatment related factors.

According to Mücke et al. most institutions in Germany irradiate with a median single dose of 1 Gy twice (40%) or three times (51%) a week and a median total dose of 6 Gy [5].

In our institutions the majority of patients received single doses of 1 Gy (83.7%). A total doses of 6 Gy was only administered in 7.4% of the cases. Most patients (54.2%) received a total dose of 4 Gy. About two thirds of our patients underwent irradiation once and one third twice a week. A limitation of our analysis is that the influence of different dose-fractionation schedules on treatment response in painful gonarthritis could not be assessed. Niewald et al. conducted a randomized trial of radiation therapy for painful heel spur, comparing a standard dose with a very low dose. Sixty-six patients were randomized to receive radiation therapy either with a total dose of 6.0 Gy applied in 6 fractions of 1.0 Gy twice weekly (standard dose) or with a total dose of 0.6 Gy applied in 6 fractions of 0.1 Gy twice weekly (low dose). After 3 months the results in the standard arm were significantly superior compared with those in the low-dose arm. The accrual of patients was stopped at this point [15]. Heyd et al. evaluated the efficacy of two different dose-fractionation schedules for radiation therapy (RT) in patients with painful heel spur. 130 patients were randomized into two groups: the low-dose (LD) group (n = 65 heels) received a total dose of 3.0 Gy given in two weekly fractions of 0.5 Gy; in the high-dose (HD) group (n = 65 heels), two weekly fractions of 1.0 Gy were applied over 3 weeks (total dose 6.0 Gy). No statistically significant difference of response to RT between both groups was observed [16]. These results are in accordance with the findings of Ott et al. who evaluated the efficacy of two different dose-fractionation schedules for radiotherapy of patients with painful elbow syndrome. One RT course consisted of 6 single fractions/ 3 weeks. Patients were randomly assigned to receive either single doses of 0.5 or 1.0 Gy. Endpoint was pain reduction. No statistically significant differences between the two single dose trial arms for early (p = 0.103) and delayed response (p = 0.246) were found [17]. Liebmann et al. explored the efficacy of low-dose radiotherapy in adjuvant induced gonarthritis in rats using different fractionation schemes to specify a possible dose and fractionation dependence. Based on the experimental data they recommended two series of 5 × 0.5 Gy with an early treatment onset and repetition in interval during the florid phase of arthritis as most effective radiotherapy regimen to prevent a full-blown arthritic reaction [18].

#### Mechanism of action

Pathophysiology of osteoarthritisis has not yet been understood completely. However, arthrosis, i.e. the degeneration of articular cartilage, leads to an inflammatory reaction in the synovial membrane which again aggravates arthrosis [19]. Several authors showed in animal models that low-dose radiotherapy attenuates the arthritic response by anti-inflammatory effects and decreases its clinical symptoms [9,18,20].

#### Risk of somatic damage and malignant transformation

The risks of radiation exposure always have to be weighed against the therapeutic benefit. Somatic damage is not expected at the given low doses [10]. Moreover since most patients are seniors damage to the genetic material plays only a minor role. According to Jansen et al. the average attributable lifetime risk for an induced fatal tumor for a 25/50/75-year-old woman is 4/1/0.5 ‰ for a double series treatment with a target dose of 12 Gy. For a single series these values are 2.0, 0.7 and 0.3 respectively [21]. Despite these considerations compliance with all health and safety rules remains a matter of course for each radiotherapist.

## Conclusions

In accordance with previous retrospective analyses, especially the large pattern of care study of the German Cooperative Group on Radiotherapy for Benign Diseases, our results confirm that low-dose radiotherapy is an effective treatment for painful osteoarthritis of the knee. The influence of radiological severity on treatment outcome remains unclear. In contrast to an earlier retrospective study we identified severe signs of osteoarthritis as positive prognostic factor for treatment response. A randomized trial is urgently required to compare radiotherapy with other therapy methods.

## Competing interests

The authors declare that they have no competing interests.

## Authors’ contributions

SK was responsible for the collection of data, statistical evaluation and writing of the manuscript. KM was responsible for the check of the data, statistical evaluation, review of the literature and writing of the manuscript. RDK, GH and DB were responsible for the conception of the study. DB and GF were responsible for the treatment of the majority of patients and the control of the documentation of the treatment and follow-up data. AL, UW and OM critically evaluated and approved the manuscript. All authors read and approved the final manuscript.

## Supplementary Material

Additional file 1: Table S1 Outcome of radiotherapy in painful gonarthritis. Overview of literature results in radiotherapy for painful gonarthritis (1980–2012) including our study, modified classification of therapy response according to von Pannewitz [22].* very good response = painless/markly improved, good/ satisfying response = improved, little/ no response = stable, ** identical patient cohort . Dose distribution in a knee (coronar view). Dose distribution in a knee with a diameter of 10 cm as a function of different irradiation techniques using an orthovoltage unit with 175 kV, 20 mA, 0.5 mm copper filter, focus-skin distance 40 cm and lateral (opposed) fields (10 cm x 15 cm). Click here for file

## References

[B1] Felson DT Osteoarthritis Rheum Dis Clin North Am 1990 16 499 512 2217954

[B2] Engelhardt M Epidemiologie der arthrose in westeuropa Dtsch Z Sportmed 2003 54 171 175

[B3] Fried G Die röntgentherapie der arthritis Strahlentherapie 1934 49 634 675

[B4] Toschke G Zur röntgenbehandlung von gelenkerkrankungen Strahlentherapie 1941 70 443 456

[B5] Mücke R Seegenschmiedt MH Heyd R Schafer U Prott FJ Glatzel M Micke O Radiotherapy in painful gonarthrosis. Results of a national patterns-of-care study Strahlenther Onkol 2010 186 7 17 10.1007/s00066-009-1995-7 20082182

[B6] Jordan KM Arden NK Doherty M Bannwarth B Bijlsma JW Dieppe P Gunther K Hauselmann H Herrero-Beaumont G Kaklamanis P EULAR recommendations 2003: an evidence based approach to the management of knee osteoarthritis: report of a task force of the standing committee for international clinical studies including therapeutic trials (ESCISIT) Ann Rheum Dis 2003 62 1145 1155 10.1136/ard.2003.011742 14644851 PMC1754382

[B7] Kellgren JH Lawrence JS Osteo-arthrosis and disk degeneration in an urban population Ann Rheum Dis 1958 17 388 397 10.1136/ard.17.4.388 13606727 PMC1007067

[B8] Von Pannewitz G Roentgen therapy for deforming arthritis 1933 Leipzig: Thieme

[B9] Fischer U Kamprad F Koch F Ludewig E Melzer R Hildebrandt G The effects of low-dose Co-60 irradiation on the course of aseptic arthritis in a rabbit knee joint Strahlenther Onkol 1998 174 633 639 10.1007/BF03038512 9879351

[B10] Keinert KSU Schumann E Ergebnisse der strahlentherapie der arthrosis deformans des kniegelenks Dt Gesundh-Wesen 1982 10 445 447

[B11] Keilholz L Seegenschmiedt H Sauer R Radiotherapy for painful degenerative joint disorders. Indications, technique and clinical results Strahlenther Onkol 1998 174 243 250 10.1007/BF03038716 9614952

[B12] Ruppert R Seegenschmiedt MH Sauer R Radiotherapy of osteoarthritis. Indication, technique and clinical results Orthopade 2004 33 56 62 10.1007/s00132-003-0568-1 14747911

[B13] Sautter-Bihl ML Liebermeister E Scheurig H Heinze HG Analgetic irradiation of degenerative-inflammatory skeletal diseases. Benefits and risks Dtsch Med Wochenschr 1993 118 493 498 10.1055/s-2008-1059354 8467752

[B14] Glatzel MF D Bäsecke S Krauß A Prognostic factors of success of analgesic radiotherapy for gonarthrosis Strahlenther Onkol 2004 180 Sondernr 1 6

[B15] Niewald M Seegenschmiedt MH Micke O Graeber S Muecke R Schaefer V Scheid C Fleckenstein J Licht N Ruebe C Randomized, multicenter trial on the effect of radiation therapy on plantar fasciitis (painful heel spur) comparing a standard dose with a very low dose: mature results after 12 months‘ follow-up Int J Radiat Oncol Biol Phys 2012 84 e455 462 10.1016/j.ijrobp.2012.06.022 22836057

[B16] Heyd R Tselis N Ackermann H Roddiger SJ Zamboglou N Radiation therapy for painful heel spurs: results of a prospective randomized study Strahlenther Onkol 2007 183 3 9 17225939 10.1007/s00066-007-1589-1

[B17] Ott OJ Hertel S Gaipl US Frey B Schmidt M Fietkau R Benign painful elbow syndrome: first results of a single center prospective randomized radiotherapy dose optimization trial Strahlenther Onkol 2012 188 873 877 10.1007/s00066-012-0179-z 22918610

[B18] Liebmann A Hindemith M Jahns J Madaj-Sterba P Weisheit S Kamprad F Hildebrandt G Low-dose X-irradiation of adjuvant-induced arthritis in rats. Efficacy of different fractionation schedules Strahlenther Onkol 2004 180 165 172 10.1007/s00066-004-1197-2 14991205

[B19] Trott KR Parker R Seed MP The effect of x-rays on experimental arthritis in the rat Strahlenther Onkol 1995 171 534 538 7570302

[B20] Hildebrandt G Jahns J Hindemith M Spranger S Sack U Kinne RW Madaj-Sterba P Wolf U Kamprad F Effects of low dose radiation therapy on adjuvant induced arthritis in rats Int J Radiat Biol 2000 76 1143 1153 10.1080/09553000050111613 10947127

[B21] Jansen JT Broerse JJ Zoetelief J Klein C Seegenschmiedt HM Estimation of the carcinogenic risk of radiotherapy of benign diseases from shoulder to heel Radiother Oncol 2005 76 270 277 10.1016/j.radonc.2005.06.034 16157402

[B22] Von Pannewitz G Röntgentherapie der arthrosis deformans Strahlentherapie 1953 92 375 382 13156820

