# Supplementary material for: Efficacy of low-dose radiotherapy in painful gonarthritis: experiences from a retrospective East German bicenter study
Source: Radiat Oncol. 2013 Jan 31;8:29. doi: 10.1186/1748-717X-8-29 (PMC3582586; doi:10.1186/1748-717X-8-29)
Supplement: Additional file 1: Table S1 — Outcome of radiotherapy in painful gonarthritis. Overview of literature results in radiotherapy for painful gonarthritis (1980–2012) including our study, modified classification of therapy response according to von Pannewitz [22].* very good response = painless/markly improved, good/ satisfying response = improved, little/ no response = stable, ** identical patient cohort . Dose distribution in a knee (coronar view). Dose distribution in a knee with a diameter of 10 cm as a function of different irradiation techniques using an orthovoltage unit with 175 kV, 20 mA, 0.5 mm copper filter, focus-skin distance 40 cm and lateral (opposed) fields (10 cm x 15 cm). [file 1748-717X-8-29-S1.doc]

## Supplementary table 1 - outcome of radiotherapy in painful gonarthritis

Overview of literature results in radiotherapy for painful gonarthritis (1980-2012) including our study, modified classification of therapy response according to von Pannewitz

| author | patients  (knees) | evaluation | positive response | painless/markly improved | improved | stable | worse |
| --- | --- | --- | --- | --- | --- | --- | --- |
| Keinert et al. 1982 | 290 | at the end of RT  6 weeks after RT | 64%  81% | 8%  38% | 56%  43% | 36%  19% | 0%  0% |
| Sautter-Bihl* et al. 1993 | 42  21 | at the end of RT  long-term | 62%  71% | 12%  14% | 50%  57% | 38%  10% | 0%  19% |
| Keilholz et al. 1998**  Ruppert et al. 2004** | (49) | 6 weeks after RT | 66% | 35% | 31% | 29% | 6% |
|  | 31 | ≥ 6 months after RT | 64% | 42% | 22% | 26% | 10% |
| Glatzel et al. 2004 | (200) | not mentioned | 68% | 37% | 31% | 32% | |
| Mücke et al. 2010 | 5069 | not mentioned | (60%) | - | - | (21%) | - |
| present study | (1659) | at the end of RT – 2 months after RT | 80% | 11% | 69% | 12% | 9% |

* very good response = painless/markly improved, good/ satisfying response = improved, little/ no response = stable, ** identical patient cohort

## Supplementary figure 1 - dose distribution in a knee (coronar view)

## Dose distribution in a knee with a diameter of 10 cm as a function of different irradiation techniques using an orthovoltage unit with 175kV, 20 mA, 0.5 mm copper filter, focus-skin distance 40 cm and lateral (opposed) fields (10cm x 15 cm)
